# Non-invasive *Streptococcus pneumoniae* infections are associated with different serotypes than invasive infections, Belgium, 2020 to 2023

**DOI:** 10.2807/1560-7917.ES.2024.29.45.2400108

**Published:** 2024-11-07

**Authors:** Ioannis Passaris, Stéphanie Depickère, Toon Braeye, Marina Mukovnikova, Alexandra Vodolazkaia, Chloé Abels, Lize Cuypers, Stefanie Desmet, Pieter-Jan Ceyssens, NIPD study group Belgium, NIPD study group Belgium, Jean-Marc Senterre, Te-din Huang, Amélie Heinrichs, Salah Lali, Philippe Lefèvre, Nélida Ciupilan, Alexandre Grimmelprez, Valérie Verbelen, Cécile Meex, Maria-Grazia Garrino, Marie Tré-Hardy, Koen Magerman, Steven Vervaeke, Kim Camps, Katelijne Floré, Louis Ide, Johan Frans, Elise Willems, Evilien Vekens, Chris Vanhentenrijk, Peter Verbeeck, Tom Spiritus, Clara Ceyssens

**Affiliations:** 1 Bacterial Diseases Unit, Sciensano, Brussels, Belgium; 2 Platform Interventional Studies, Sciensano, Brussels, Belgium; 3 Epidemiology of Infectious Diseases, Sciensano, Brussels, Belgium; 4 Laboratory of Medical Microbiology, Sciensano, Brussels, Belgium; 5 MSD Belgium, Brussels, Belgium; 6 National Reference Centre for invasive *Streptococcus pneumoniae*, UZ Leuven, Leuven, Belgium; 7 Laboratory of Clinical Microbiology, Department of Microbiology, Immunology and Transplantation, KU Leuven, Leuven, Belgium; 8 The members of the NIPD study group Belgium are listed under Collaborators

**Keywords:** *Streptococcus pneumoniae*, non-invasive pneumococcal disease, serotype, antimicrobial resistance, protein conjugate vaccines

## Abstract

**Background:**

Despite widely implemented pneumococcal vaccination programmes, *Streptococcus pneumoniae* remains a global risk for human health. *Streptococcus pneumoniae* can cause invasive (IPD) or non-invasive pneumococcal disease (NIPD). Surveillance is mainly focusing on IPD, assessing the full impact of pneumococcal vaccination programmes on pneumococcal disease is challenging.

**Aim:**

We aimed to prospectively investigate serotype distribution and antimicrobial resistance (AMR) of *S. pneumoniae* isolates from patients with NIPD and compare with data on IPD isolates and with a 2007–2008 dataset on NIPD.

**Methods:**

Between September 2020 and April 2023, we collected isolates and patient data from patients with NIPD from 23 clinical laboratories in Belgium. Capsular typing was performed by a validated Fourier-Transform Infrared spectroscopic method, and AMR was assessed with broth microdilution, using the European Committee on Antimicrobial Susceptibility Testing (EUCAST) clinical breakpoints.

**Results:**

We received *S. pneumoniae* isolates from 1,008 patients with lower respiratory tract infections (n = 760), otitis media (n = 190) and sinusitis (n = 58). Serotype 3 was the most prevalent serotype among the NIPD isolates. Serotypes not included in the 20-valent pneumococcal conjugate vaccine (PCV20) were significantly more common among the NIPD than among the IPD isolates. Antimicrobial resistance levels were significantly higher among the NIPD isolates (n = 539; 2020–2022) compared with the IPD isolates (n = 2,344; 2021–2022). Resistance to several β-lactam antimicrobials had increased significantly compared with 15 years before.

**Conclusions:**

The NIPD isolates were strongly associated with non-vaccine serotypes and with increased AMR levels. This underlines the importance of continued NIPD surveillance for informed policy making on vaccination programmes.

**Key public health message**

**What did you want to address in this study and why?**

Vaccines against pneumococcal disease protect against 13–23 variants of *Streptococcus pneumoniae*. Vaccination programmes are predominantly based on findings of *S. pneumoniae* from blood and other invasive infections. We aimed to investigate *S. pneumoniae* from respiratory infections (non-invasive) and compare them with isolates from invasive infections in Belgium.

**What have we learnt from this study?**

About 25% of *S. pneumoniae* isolates from respiratory infections belonged to variants included in the pneumococcal vaccines. Some *S. pneumoniae* variants were more likely to cause either respiratory or invasive infections. Antimicrobial resistance levels were higher among *S. pneumoniae* isolates from respiratory infections than among isolates from invasive infections. Antimicrobial resistance has increased compared with 2007–2008.

**What are the implications of your findings for public health?**

As respiratory infections have a high burden and are more common than invasive infections, *S. pneumoniae* isolates from respiratory infections should be routinely monitored. Variants of *S. pneumoniae* from respiratory infections should be considered when pneumococcal vaccines are selected. Our data can form the basis for cost-effectiveness calculations for decisions on pneumococcal vaccination programmes.

## Introduction

Infections of the opportunistic, Gram-positive pathogen *Streptococcus pneumoniae* caused over 500,000 deaths in 2019 globally [1]. Most pneumococcal strains are encapsulated, and the capsule forms the target of all currently licensed vaccines [2]. Invasive *S. pneumoniae* infections (IPD) occur in normally sterile sites in the human body, including blood, cerebrospinal fluid and pleural fluids [3]. The inclusion of pneumococcal conjugate vaccines (PCVs) in childhood vaccination programmes 2000–2010 led to an initial decline in IPD incidence in children and to a lesser extent in adults (herd protection effect) [4]. This decline has recently stagnated, and IPD incidence has started to increase again in Belgium and other countries due to (i) persistent circulation of serotypes included in vaccines, such as serotypes 3 and 19A, sometimes driven by changes in the vaccination programmes and (ii) circulation of serotypes not covered by vaccines [5-7].

Non-invasive pneumococcal disease (NIPD) affects the upper and lower respiratory tracts, presenting typically as community-acquired pneumonia, otitis media (OM) or sinusitis [8]. Although IPD is associated with more severe outcomes, NIPD is more common, and mortality rates of community-acquired pneumonia can range from 6.4% to > 40%, depending on the medical care setting [9]. Despite the disease burden of NIPD, surveillance programmes and studies of NIPD are generally lacking. More recently, small-scale surveys have been performed on serotype distribution and antimicrobial resistance (AMR) of pneumococci from patients with NIPD [10-12]. The limited data available on NIPD hint at a similar persistence of specific vaccine-type serotypes and serotype replacement as for IPD, but detailed time series analyses are lacking.

In Belgium, pneumococcal vaccination for children is voluntary and free of charge. Prevnar, a 7-valent pneumococcal conjugate vaccine (PCV7) including serotypes 4, 6B, 9V, 14, 18C, 19F and 23F, was introduced into the vaccination programme for children in 2007, after which vaccination rates quickly reached > 90%. In 2011, PCV7 was replaced by Prevnar 13, a 13-valent PCV (PCV13) including the PCV7 serotypes and serotypes 1, 3, 5, 6A, 7F and 19A. Between 2015 and 2016, Belgium switched from PCV13 to Synflorix, a 10-valent PCV (PCV10) which includes the PCV7 serotypes and serotypes 1, 5 and 7F. This decision was reconsidered in 2019 after a significant rise in children IPD caused by serotype 19A, which is present in PCV13, but not in PCV10 [7]. Since 2023, parents have had the choice between PCV13 or Vaxneuvance, a 15-valent PCV (PCV15), which includes PCV13 serotypes and serotypes 22F and 33F, but only PCV13 is free of charge. Vaccination of adults is recommended for people of certain age and risk groups, but currently only Apexxnar, a 20-valent PCV (PCV20), which includes PCV15 serotypes and serotypes 8, 10A, 11A, 12F and 15B, is reimbursed for adults aged 65–80 years with one or more comorbidities and not previously vaccinated with Pneumovax 23, a pneumococcal polysaccharide vaccine (PPV23) including serotypes 1, 2, 3, 4, 5, 6A, 7F, 8, 9N, 9V, 10A, 11A, 12F, 14, 15B, 17F, 18C, 19A, 19F, 20, 22F, 23F and 33F. Since August 2022, the recommended vaccine for this group is PCV20, or as an alternative, PCV15 administration is proposed followed by PPV23 8 weeks–1 year later (depending on the specific age and risk group). Nonetheless, uptake has been consistently low, 21–32% [13,14].

The National Reference Centre (NRC) of invasive *S. pneumoniae* at the University hospital in Leuven (UZ Leuven), coordinates a passive laboratory surveillance network of Belgium. The NRC has monitored the epidemiology of IPD in Belgium for more than 20 years and is crucial in informing policymakers on implementing and sustaining pneumococcal vaccination programmes [15]. In 2020, the Belgian Nasopharyngeal (NP) carriage Study Group on pneumococcal colonisation in (healthy) children observed a significant rise in serotype 19A carriage in children aged < 2.5 years attending nurseries, yielding important information on transmission dynamics of *S. pneumoniae* serotypes in Belgium [16]. Between 2016 and 2018, almost 80% of Belgian children in preschools and nurseries carried *S. pneumoniae*, and serotypes 6C, 23B, 11A and 15B, which are not covered by PCV13 or PCV15, were most common [17]. Moreover, some serotypes were found more often in samples from children with IPD [18] or acute otitis media (AOM) [19] than in samples from children without symptoms of (respiratory) illness, suggesting that certain serotypes are more likely to cause disease than others.

In Belgium, as in many other countries, we have very limited information on the serotype distribution of NIPD isolates. This information is nonetheless relevant for the evaluation of the impact of the vaccination programmes on pneumococcal diseases [20], especially with the higher valency vaccines (Vaxneuvance [21] and Apexxnar [22]) currently on the market and new vaccines in the pipeline [23-26]. It is unclear if vaccination of adults could considerably decrease the incidence of vaccine-preventable NIPD and if the intervention is cost-effective for it to be included in the governmental vaccination programmes [27]. We aimed to conduct a prospective study to analyse non-invasive *S. pneumoniae* isolates from routine hospital settings in Belgium during a 32-month period and compare with IPD isolates from the passive surveillance.

## Methods

### Study design

A total of 23 Belgian clinical laboratories agreed to send non-invasive *S. pneumoniae* isolates to Sciensano, Brussels, Belgium between September 2020 and April 2023. A full list of the participating laboratories and their geographical location is shown in Supplementary Figure S1. The inclusion criteria were: (i) patients diagnosed with a lower respiratory tract infection (LRTI), OM or sinusitis and (ii) *S. pneumoniae* isolated from the upper (e.g. pus or fluid from nasal sinus, fluid from middle ear or a swab sample) or lower (e.g. sputum, bronchial or endotracheal aspirate, bronchoalveolar fluid) respiratory tract. The exclusion criteria were: (i) *S. pneumoniae* isolated from blood or another usually sterile specimen and (ii) another cause of disease. If more than one *S. pneumoniae* isolate was obtained from the same patient during the study, only the first was included, but if two different serotypes were simultaneously detected in one patient, the two isolates were included for typing. In addition to the isolates, we collected information on the isolation date, specimen type, sex, age, type of medical care, clinical diagnosis, comorbidities or weakened immune system, vaccination status and detection of other respiratory pathogens of the patient. The study was registered with ClinicalTrials.gov (NCT04447521).

### Microbiological analyses

At Sciensano, the species identification of *S. pneumoniae* isolates was confirmed using optochin (optochin disks, Merck, Darmstadt, Germany) sensitivity and bile solubility assays (sodium deoxycholate, Merck) [28,29], supplemented by matrix assisted laser desorption ionization (MALDI-TOF) (Bruker, Billerica, the United States (US)) in case of inconsistency. Antimicrobial susceptibility of the first 539 isolates was tested on customised Sensititre plates (Thermo Scientific, Waltham, US), following the supplier’s instructions and using the European Committee on Antimicrobial Susceptibility Testing (EUCAST) clinical breakpoints version 13.0 [30]. The tested antimicrobials and the concentration ranges were: tetracycline (0.25–8 mg/L), penicillin (0.015–8 mg/L), levofloxacin (0.008–4 mg/L), cefotaxime (0.015–4 mg/L), clindamycin (0.015–4 mg/L), meropenem (0.015–2 mg/L), cefuroxime (0.5–8 mg/L), moxifloxacin (0.004–4 mg/L), erythromycin (0.25–4 mg/L), amoxicillin (0.12–4 mg/L), imipenem (0.12–4 mg/L), trimethoprim-sulfamethoxazole (0.06/1.19–2/38 mg/L) and amoxicillin-clavulanic acid 2:1 ratio (2:1–16:8 mg/L).The AMR data extracted from NIPD isolates in Belgium, 2007–2008 [31], were reanalysed using the current EUCAST clinical breakpoints.

After species identification, the isolates were stored in Cryotube vials (Sarstedt, Nümbrecht, Germany) at −80°C. For capsule typing, bacterial material from the Cryotube was inoculated with a 10 µL inoculation loop (Sarstedt) onto Columbia (CB) agar supplemented with  5% sheep blood (Thermo Scientific) and incubated at 36°C in 5% CO_2_ for 24 h. The capsule type was determined by Fourier Transform Infrared (FT-IR) spectroscopy (IR Biotyper, Bruker) using a previously validated method [32]. Briefly, serotypes of the pneumococcal isolates were determined by comparing an average spectrum from at least three technical replicates to an in-house reference database. This calculation was performed by a combination of a machine learning algorithm (PneumoClassifier) and hierarchical cluster analysis (applied on spectra that underwent dimensionality reduction analysis) with a dendrogram as output. However, serogroup 24 serotypes (24A, 24B, 24C and 24F) cannot be discriminated from each other and from serotype 40 with this method, neither can serotype 33F and 33A and serotype 29 and serotype 35D be accurately discriminated. Therefore, in this article, isolates belonging to one of these serotypes are grouped as serogroup 24/40, serotype 33F/33A or serotype 29/35D. Data on isolates from patients with IPD were provided by the NRC of invasive *S. pneumoniae* at UZ Leuven [7,33].

### Statistical analyses

First, an analysis of the association between patient age group and (i) clinical diagnosis, (ii) type of medical care, (iii) comorbidities or weakened immune system, (iv) patient sex and (v) geographical region, as well as between patient sex and comorbidities or weakened immune system was conducted using Pearson’s chi-square test and Fisher’s exact tests, considering a p value of < 0.05 as statistically significant. Bonferroni corrections were applied for multiple testing. All distributions were expressed as proportions (%), and 95% confidence intervals (CI) were calculated with the binomial exact method. Next, the association between serotype (or serotype cohort when serotypes were grouped according to their inclusion in PCVs) and (i) clinical diagnosis, (ii) patient age group and (iii) IPD/NIPD was analysed using the same methods. Similarly, Fisher’s exact tests were used to compare AMR levels between IPD and NIPD and between different time periods for the different antibiotics. Predicted probabilities of receiving a specific clinical diagnosis or type of medical care by patient age group were obtained from multinomial logistic regression models. Finally, to investigate the association between serotype and IPD or NIPD, we performed a logistic regression. We included data from 2021 and 2022 on serotype, calendar time, sex and age of the patient. The final model included age as a spline with knots on 1 and 65 years and serotype as a factor, with serotype 15A as the reference level. Serotypes found in fewer than 10 cases and serotypes not typeable with FT-IR spectroscopy were excluded. Following the model fit, age-adjusted relative risks (aRR) were obtained by calculating the ratio of the predicted probability of IPD given *serotype_i_ P(IPD│serotype_i_)*, to the probability over the other serotypes *P(IPD│serotype_#i_)*. The 95% confidence intervals for the aRR were established through bootstrapping.

Analyses were done in R (https://www.r-project.org/) and all graphs were made with GraphpadPrism 9.4 (https://www.graphpad.com).

## Results

### Characteristics of patients with non-invasive *Streptococcus pneumoniae* infection

**Figure 1:**
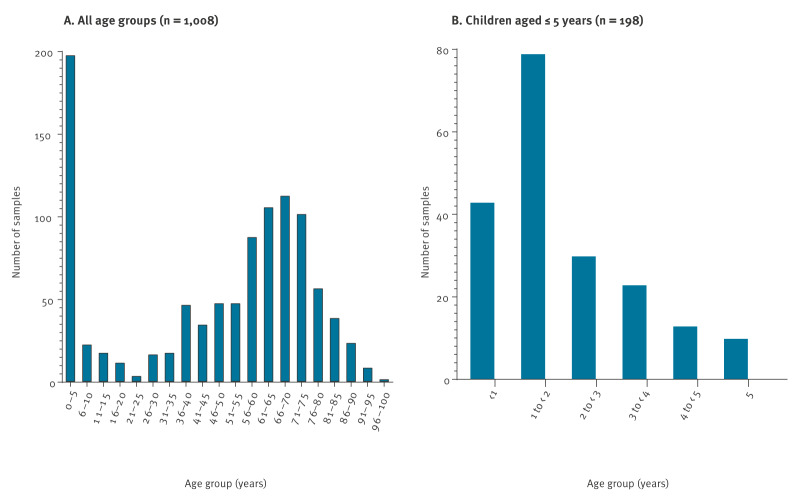
Age distribution of patients with non-invasive *Streptococcus pneumoniae* infection, Belgium, September 2020–April 2023 (n = 1,008)

We included isolates from 1,008 patients with non-invasive *S. pneumoniae* infections (Table). Almost half of these patients (n = 484; 48%) received ambulatory care, 427 (42.4%) were hospitalised and 95 (9.4%) were admitted to an intensive care unit. Also, 198 (19.6%) patients were children aged < 5 years and 409 (40.6%) adults aged 56–75 years (Figure 1A). More than half of the patients (n = 122; 61.6%) in the 0–5-year age group were aged < 2 years (Figure 1B). Results on the association of age with other patient characteristics (univariate analysis and logistic regression) are presented in Supplementary Figures S2–S3.

**Table:** Demographic and clinical characteristics of patients with non-invasive *Streptococcus pneumoniae* infection, Belgium, September 2020–April 2023 (n = 1,008)

Characteristics	Number	Percentage
Number of patients with more than one *Streptococcus pneumoniae* isolate	15	1.5
Region		
Wallonia	603	59.8
Flanders	398	39.5
Brussels	7	0.7
Age (years)		
< 16	239	23.7
16–49	177	17.6
50–64	224	22.2
> 64	368	36.5
Sex		
Male	572	56.7
Female	435	43.2
Unknown	1	0.1
Type of medical care		
Ambulatory care	484	48.0
Hospital admission	427	42.4
Intensive care unit	95	9.4
Long-term care facility	1	0.1
Unknown	1	0.1
Comorbidities or weakened immune system		
Patients with at least one comorbidity or weakened immune system	391	38.8
Chronic obstructive pulmonary disease	203	20.1
Cancer	53	5.3
Diabetes	47	4.7
Other pathogens identified		
Specimens with at least one more viral or bacterial pathogen detected	419	41.2
*Haemophilus influenzae*	247	24.5
SARS-CoV-2	52	5.2
Specimen type		
Sputum	517	51.3
Middle ear fluid	173	17.2
Endotracheal or bronchial aspiration	152	15.1
Bronchoalveolar lavage	65	6.4
Sinus aspirate	38	3.8
Nasopharyngeal aspirate or swab	20	2.0
Nasal swab	15	1.5
Respiratory pus	10	1.0
Pus (ear)	7	0.7
Other	11	1.1
Clinical diagnosis		
Lower respiratory tract infection	760	75.4
Otitis media	190	18.8
Sinusitis	58	5.8
Vaccination status		
Unknown	783	77.7
Not vaccinated	126	12.5
Vaccinated	99	9.8
Vaccine (n = 99)		
Prevnar 13 (PCV13)	42	42.4
Pneumovax 23 (PPV23)	21	21.2
Synflorix (PCV10)	5	5.1
Prevnar 13 and Pneumovax 23	1	1.0
Unknown vaccine	30	30.3

SARS-CoV-2: severe acute respiratory syndrome coronavirus 2.

### *Streptococcus pneumoniae* serotypes of non-invasive infections

**Figure 2:**
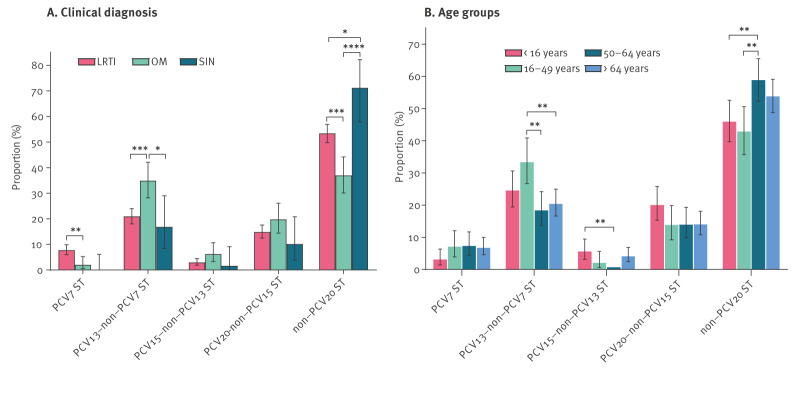
Serotypes of *Streptococcus pneumoniae* isolates from patients with non-invasive infections, by clinical diagnosis and age, Belgium, September 2020–April 2023 (n = 1,023)

LRTI: lower respiratory tract infection; OM: otitis media: PCV: pneumococcal conjugate vaccine; PCV7: pneumococcal 7-valent conjugate vaccine (serotypes 4, 6B, 9V, 14, 18C, 19F and 23F); PCV13: pneumococcal 13-valent conjugate vaccine (PCV7 serotypes and serotypes 1, 3, 5, 6A, 7F and 19A); PCV15: pneumococcal 15-valent conjugate vaccine (PCV13 serotypes and serotypes 22F and 33F); PCV20: pneumococcal 20-valent conjugate vaccine (PCV15 serotypes and serotypes 8, 10A, 11A, 12F and 15B); SIN: sinusitis; ST: serotype; * : p < 0.05; **: p < 0.01; *** : p < 0.001 and ****: p < 0.0001.

We serotyped 1,023 isolates from 1,008 patients, 15 patients had more than one isolate. Serotype 3 was most common (n = 170; 16.6%), followed by 11A (n = 80; 7.8%), 6C (n = 79; 7.7%), 23B (n = 74; 7.2%) and 19A (n = 66; 6.5%). When the serotypes were grouped according to their presence in one of the currently licensed PCVs and the clinical diagnosis (LRTI, OM, sinusitis), significant differences were found within the PCV7, PCV13-nonPCV7 and non-PCV20 serotypes (Figure 2A). Serotype 3, which is included in PCV13 and later vaccines but not in PCV7 or PCV10, was identified in 49 (28.6%) patients diagnosed with OM, whereas serotypes 19F (included in all vaccines) and 6C, 15A and 35B (not included in any licensed vaccines) were found more often in patients with LRTI than in patients with OM. The difference, though not significant, between the PCV7 serotypes was largely driven by 19F, while within the PCV20 serotypes the statistically significant difference (p < 0.001) was driven by serotype 6C and further amplified by serotypes 15A, 23A and 35B. The list of serotypes is presented in Supplementary Table S1 and a more detailed analysis on the specific serotypes is presented in Supplementary Figure S4.

Panel A. Number of isolates included: LRTI (n = 772); OM (n = 192) and sinusitis (n = 59).

Panel B. Number of isolates included: < 16 years (n = 243); 16–49 years (n = 179); 50–64 years (n = 227); > 64 years (n = 374).

Statistical significance was assessed with a chi-square test and exact Fisher’s tests were performed for pairwise comparisons (a Bonferroni correction for multiple testing was applied). Error bars represent 95% confidence intervals calculated by the binomial exact method.

Serotype 3 was found more often in patients aged 16–49 years than in patients aged ≥ 50 years (p < 0.01) (Figure 2B). Serotype 3 was also significantly more associated with patients with no comorbidities or weakened immune system (n = 95; 18.8%) than with those with these conditions (n = 44; 11.0%; p < 0.01). Serotypes 22F and 33F, included in PCV15 and later vaccines but not in earlier vaccines, were more common in patients aged ≤ 16 years (n = 198; 81.5% children in this age group were aged ≤ 5 years) than in patients aged 50–64 years (Figure 2B). Serotypes 6C, 15A, 23A, 31 and 35B, not included in any vaccines, were found more often in patients aged ≥ 50 years (n = 136, 22.6%) than in the younger age groups (n = 49, 11.6%; p <0.0001). The proportions of the various serotypes are depicted in Supplementary Figure S4B.

### Antimicrobial susceptibility testing of *Streptococcus pneumoniae* from non-invasive infections

**Figure 3:**
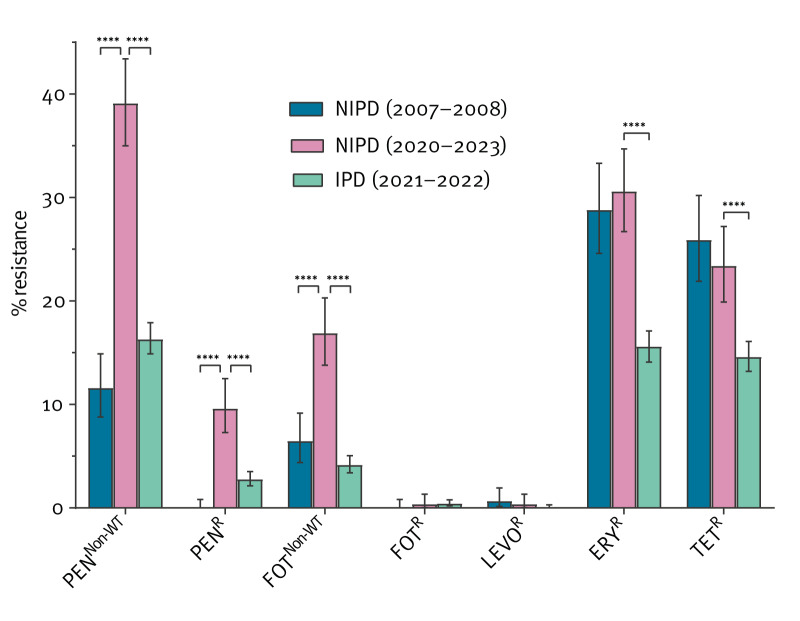
Antimicrobial resistance of *Streptococcus pneumoniae* isolates from patients with invasive infections, 2021–2022 (n = 2,344) and non-invasive infections, 2007–2008 (n = 448) and 2020–2022 (n = 539), Belgium

ERY: erythromycin; FOT: cefotaxime; IPD: invasive pneumococcal disease; LEVO: levofloxacin; MIC: minimum inhibitory concentration; NIPD: non-invasive pneumococcal disease; PEN: penicillin; R: resistant; TET: tetracycline; WT: wild-type; * = p < 0.05; ** = p < 0.01; *** = p < 0.001; **** = p < 0.0001.

Of the 539 NIPD isolates, 211 (39.1%) had non-wild type resistance to penicillin (minimum inhibitory concentration (MIC) > 0.06 mg/L). Full penicillin resistance (10%; MIC > 2 mg/L) was more often observed in isolates of serotypes 11A (21/53) and 19F (5/26). Likewise, non-wild type resistance to cefotaxime (17%; MIC > 0.5 mg/L) was largely seen in serotypes 19F (11/26), 11A (22/53), 15A (6/20), 15B (2/10) and 35B (5/18), while full cefotaxime resistance was rare (0.6%; MIC > 2 mg/L) and observed in isolates from serotypes 9N (1/18), 23B (1/48) and 35B (1/18). Compared with a previous study on Belgian NIPD from the period 2007–2008 [31], AMR levels to penicillin and cefotaxime increased significantly while resistance to levofloxacin, erythromycin and tetracycline was largely unchanged (Figure 3). Furthermore, MIC90 levels of penicillin, cefotaxime, amoxicillin and imipenem have increased, as presented in Supplementary Table S3. Isolates from patients with NIPD were more resistant to penicillin, cefotaxime, erythromycin and tetracycline than isolates from patients with IPD, sampled 2021–2022 (Figure 3). More detailed results of the susceptibility testing are presented in Supplementary Table S2.

Exact Fisher’s tests were performed to assess differences between the NIPD 2020–2023 population and the other two populations (a Bonferroni correction for multiple testing was applied). Error bars represent 95% confidence intervals calculated by the binomial exact method.

Penicillin: non-wild type: MIC > 0.06 mg/L, resistant: MIC > 2 mg/L); cefotaxime: non-wild type: MIC > 0.5 mg/L, resistant: MIC > 2 mg/L); levofloxacin: resistant: MIC > 2 mg/L; erythromycin: resistant: with MIC > 0.5 mg/L; tetracycline: resistant: MIC > 2 mg/L.

### Comparison of *Streptococcus pneumoniae* isolates from patients with invasive and non-invasive infections

**Figure 4:**
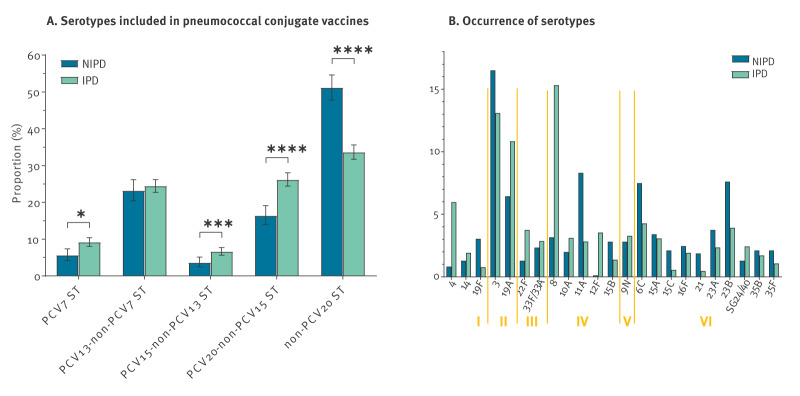
Serotypes of *Streptococcus pneumoniae* isolates from patients with invasive (n = 2,344) and non-invasive (n = 854) infections, Belgium, 2021–2022^a^

**Figure 5:**
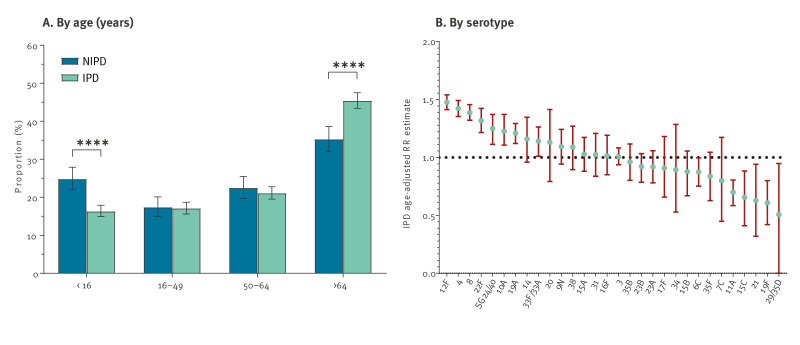
Age distribution and serotypes of patients with invasive (n = 2,344) and non-invasive (n = 854) *Streptococcus pneumoniae* infection, Belgium, 2021–2022^a,b^

IPD: invasive pneumococcal disease; NIPD: non-invasive pneumococcal disease; RR: relative risk; * = p < 0.05; ** = p < 0.01; *** = p < 0.001; **** = p < 0.0001.

^a^ Information on age was missing from nine isolates.

^b^ Only serotypes with a proportion of > 1.5% in IPD or NIPD are included in the figure.

We received data on 2,344 *S. pneumoniae* isolates from patients with IPD, sampled between 2021 and 2022 and compared the IPD isolates [33] with 854 isolates from patients with NIPD, sampled between 2021 and 2022. Serotypes 4, 19A, 22F, 8 and 12F, included in the PCV20 vaccine, were more common in patients with IPD (n = 924, 39.4%) than in those with NIPD (n = 101, 11.8%) (Figure 4). On the other hand, serotypes not included in PCV20 vaccines were more common in NIPD (n = 213; 24.9%) than in IPD (n = 296; 12.6%). In total, PCV20 serotypes were isolated in 66.4% of IPD patients and in 48.8% of NIPD patients. Younger patients (< 16 years) had more often NIPD, and older patients (> 64 years) more often IPD (Figure 5A). As some serotypes were more often identified in patients of certain age groups, age could be a potential confounder, thus we calculated age-adjusted relative risk (aRR) estimates (Figure 5B). When we compared with all serotypes, aRR estimates > 1 corresponded to serotypes associated with IPD, while aRR estimates < 1 to serotypes with NIPD. Serotypes 12F (aRR = 1.48; 95% CI: 1.41–1.54), 4 (aRR = 1.42; 95% CI: 1.35–1.49), 8 (aRR = 1.39; 95% CI: 1.32–1.46), 22F (aRR = 1.32; 95% CI: 1.22–1.42), serogroup 24/40 (aRR = 1.25; 95% CI: 1.11–1.37), 10A (aRR = 1.23; 95% CI: 1.11–1.37) and 19A (aRR = 1.21; 95% CI: 1.12–1.29) were significantly more often associated with IPD, and serotypes 29/35D (aRR = 0.51; 95% CI: 0.0000004–0.95), 19F (aRR = 0.61; 95% CI: 0.42–0.8), 21 (aRR = 0.63; 95% CI: 0.32–0.94), 15C (aRR = 0.66; 95% CI: 0.41–0.88) and 11A (aRR = 0.7; 95% CI: 0.58–0.81) with NIPD.

IPD: invasive pneumococcal disease; NIPD: non-invasive pneumococcal disease; PCV: pneumococcal conjugate vaccine; PCV7: pneumococcal 7-valent conjugate vaccine (serotypes 4, 6B, 9V, 14, 18C, 19F and 23F); PCV13: pneumococcal 13-valent conjugate vaccine (PCV7 serotypes and serotypes 1, 3, 5, 6A, 7F and 19A); PCV15: pneumococcal 15-valent conjugate vaccine (PCV13 serotypes and serotypes 22F and 33F); PCV20: pneumococcal 20-valent conjugate vaccine (PCV15 serotypes and serotypes 8, 10A, 11A, 12F and 15B); PPV: pneumococcal polysaccharide vaccine; PPV23: 1, 2, 3, 4, 5, 6A, 7F, 8, 9N, 9V, 10A, 11A, 12F, 14, 15B, 17F, 18C, 19A, 19F, 20, 22F, 23F and 33F; * = p < 0.05; ** = p < 0.01; *** = p < 0.001; **** = p < 0.0001.

^a^ Only serotypes with a proportion of > 1.5% in IPD or NIPD are included in the figure.

Panel B. The vertical yellow lines demarcate the serotypes included in different PCVs: I: PCV7; II: PCV13-non-PCV7; III: PCV15-non-PCV13; IV: PCV20-non-PC15; V: PPV23; VI: non-PCV20.

Statistical significance was assessed with a chi-square test and exact Fisher’s tests were performed for pairwise comparisons (p values < 0.05 were considered statistically significant). Error bars represent 95% confidence intervals calculated by the binomial exact method.

Panel A. Error bars represent 95% confidence intervals calculated by the binomial exact method.

Panel B. Error bars represent 95% confidence intervals calculated by bootstrapping.

aRR estimates > 1 point to serotypes that are more associated with IPD than NIPD. Statistical significance was assessed with a chi-square test and exact Fisher’s tests were performed for pairwise comparisons.

## Discussion

We identified significant differences in serotype distributions and AMR levels between isolates of *S. pneumoniae* from patients with IPD and NIPD. In our study, serotypes included in PCV20 were found in 48.8% of the NIPD isolates and in 66.4% of the IPD isolates. The current vaccines have been specifically developed to control serotypes with a high IPD disease burden, thus it is not surprising to find a high proportion of PCV20 serotypes among IPD isolates, as shown also in previous studies [30,34,35]. We demonstrated that, at least in Belgium, PCV20 (and PCV15) may have a lower impact on NIPD prevention than on IPD prevention when solely looking at serotype coverage. However, it is important to note that the protective effect of PCVs on NIPD, and more specifically on OM, goes beyond preventing only vaccine-type disease. Notably, early prevention of acute OM episodes with vaccine-type serotypes might reduce subsequent episodes of complex OM by non-vaccine serotypes and pathogens other than *S. pneumoniae* [36].

Obtaining a high serotype coverage is important for new vaccine candidates. A 21-valent vaccine named V116 has eight serotypes not included in any other PCV (serotype 15A, 15C, 16F, 23A, 23B, 24F, 31 and 35B) and was specifically designed to counter residual IPD in adults [26]. This vaccine could cover 69.2% (or 79.5% when including cross-protection to serotype 15B and 6C) of NIPD infections and 77.9% (or 83.5% when including cross-protection to serotype 15B and 6C) of IPD infections in Belgium, thus achieving substantially higher serotype coverage than PCV20 for NIPD and IPD [37]. Thus, V116 could become an interesting future alternative for countering adult pneumococcal disease. However, most of the serotype distribution dynamics observed are the result of the direct and indirect (herd protection) effect of the vaccination programme for children, but vaccines for adults may only have a limited effect on the serotype distribution in children. Future surveillance programmes in countries with vaccination programmes for children and adults should aim at including both children and adults in their study groups when determining vaccine impact.

Serotype 3 (included in PCV13) was the most common serotype (16.6%) among the NIPD isolates: 25% of the 16–49-year-olds and patients with OM (28.6%) had this serotype. Otitis media is diagnosed predominantly in children (84.2% of OM cases in our study were diagnosed in children aged < 16 years) and given the high vaccination rate of > 90% in Belgium, they have most probably been (partially) vaccinated with PCV13 (or PCV10 when born between 2015 and 2019) and thus directly protected against serotype 3 disease (when vaccinated with PCV13). However, serotype 3 is able to evade antibody-mediated clearance through its thick mucoid capsule and its capacity to shed it in specific circumstances [38]. Even though clinical studies have shown PCV13 to be effective in preventing serotype 3 pneumococcal disease [5], albeit to a lesser extent compared with other PCV13 serotypes, this serotype keeps circulating and causing disease globally. Two other PCV13 serotypes, serotypes 19A and 19F, were found in 6.5% and 3.6% of the NIPD isolates, respectively, in our study. This persistent circulation might be linked to the extensive genetic diversity within this serogroup (particularly for serotype 19A) and with increased resistance to complement deposition and lower sensitivity to opsonophagocytic killing (particularly for serotype 19F) [39,40].

Serotypes 11A (7.8%), 6C (7.7%) and 23B (7.2%) were the three most common serotypes among the NIPD isolates not included in PCV13. However, serotype 6C could be categorised as a vaccine-related serotype due to cross-protection with serotype 6A antigen included in PCV13 [37]. Of these three serotypes, only 11A is included in PCV20. Although these serotypes were found at similar proportions among the NIPD isolates, serotype 6C was more often causing LRTI and sinusitis compared with OM while serotype 23B was more often causing sinusitis compared with LRTI and OM. Interestingly, nasopharyngeal carriage data from Belgian preschool children (aged 6–30 months) in the period 2016–2019 showed high prevalence of these serotypes [17,19]. Pneumococcal carriage in children is the driver of pneumococcal transmission in the population [41,42]. Therefore, dominant serotypes in children could be expected to be the same as in the patients with NIPD. Indeed, serotypes associated with carriage and NIPD are considered better adapted to long(er) residence times in the human host, while serotypes associated with invasive disease have evolved to more efficiently enter normally sterile sites such as blood and cerebrospinal fluid [43-45].

The comparison between NIPD and IPD serotyping data showed serotypes with a predilection for causing IPD (serotype 12F, 4, 8, 22F, SG24/40, 10A and 19A), while others were more associated with NIPD (serotype 29/35D, 19F, 21, 15C and 11A). Our results align with the results of the studies in Sweden, Portugal, and Spain, in particular for IPD-associated serotypes 12F and 8 and for NIPD-associated serotype 11A, suggesting that the predilection for IPD/NIPD could, at least partly, be universal [11,46,47]. Moreover, serotypes found in asymptomatic Belgian children in the 2015–2018 period [18] corresponded fairly well with the NIPD-associated serotypes of our study, suggesting that the serotypes in asymptomatic children and patients with NIPD are more similar than the serotypes identified from patients with IPD. Some serotypes are more prone to cause IPD than others (i.e. have a higher invasive potential) but the exact genetic determinants that might explain this are still largely unknown [34,43,47]. The capsule itself is an important virulence determinant, together with other factors that dynamically remodel the capsule [48-53]. However, other genetic determinants may also play a role, as certain genetic lineages have higher invasive potentials than others, while expressing the same capsule structures [54-57]. We did not study genetic lineages, thus, future genomic studies will help to further unravel the observed differences between the IPD and NIPD isolates.

In our study, as in an earlier Australian study, isolates from non-invasive disease had an increased resistance to β-lactams compared with isolates from invasive disease [57]. We also observed an increase in non-susceptibility and resistance against penicillin among the NIPD isolates when compared with 2007–2008. This observation is worrisome as β-lactams are still the first line antimicrobials used for treatment of pneumococcal infections [3]. It is possible that PCV vaccinations, in combination with the continued high use of specific antimicrobials, such as amoxicillin, in Belgium [58], have enabled certain penicillin-resistant *S. pneumoniae*, of serotypes not included in PCV13, to thrive (such as serotype 11A isolates). The MIC50 values for penicillin and cefotaxime between our study and the 2007–2008 study were at comparable levels, whereas we observed an increase in the MIC90 values for penicillin, amoxicillin, cefotaxime and imipenem. This may worryingly mean that higher concentrations of antimicrobials are now required to inhibit the growth of β-lactam-resistant *S. pneumoniae* associated with NIPD. Isolates from patients with NIPD were more resistant to penicillin, cefotaxime, erythromycin and tetracycline than those from IPD patients. A possible explanation for this difference could be the different serotype distributions between the two populations, as certain serotypes are more often associated with AMR than others [59,60]. In this dataset, isolates belonging to serotypes 19F, 11A, 6C and 15C were associated with increased resistance.

One of the key limitations of this study is the clinical diagnosis of non-invasive pneumococcal infections. We cannot rule out that some samples may have originated from persons with pneumococcal carriage rather than true pneumococcal infections. Moreover, detection of other respiratory pathogens in the clinical specimens was common (in around 40% of specimens more than one pathogen was detected), and this exemplifies the complexity when interpreting NIPD data. We can, thus, not exclude that other respiratory pathogens may have contributed to the disease or might have been the causative agent of the illnesses. Furthermore, as the participating centres applied different clinical procedures, it was challenging to accurately harmonise the diagnostic criteria, and we needed to merge all lower respiratory tract infections into one group. In our study, serotyping was performed using FT-IR spectroscopy, while IPD serotyping data were obtained using the Quellung reaction. We demonstrated in a previous validation study, however, that the FT-IR spectroscopy had a good concordance with the Quellung reaction, strengthening our consideration that the serotyping data are comparable [32]. In our study, patients were enrolled between 2020 and 2023 which coincided with the immediate post-PCV10 and early PCV13 period in the Belgian vaccination programme for children. During the PCV10 period, steep increases of the proportion of serotype 19A and 6C were seen in the children with IPD and carriage [7,17], and these serotypes are known to be associated with increased AMR [60,61]. It is thus possible that the AMR levels reported in this study will taper off in the near future in response to the fully matured PCV13 vaccination programme for children. Finally, the recruitment period for our study overlapped with the COVID-19 pandemic, and mitigation measures taken to control the pandemic decreased the incidence of many respiratory pathogens, such as pneumococci [62]. The impact of the mitigation measures on the NIPD serotype distribution in Belgium is not well known as pre-pandemic NIPD data are scarce.

## Conclusion

Pneumococcal diseases still have a high burden on our society, despite childhood vaccination programmes implemented worldwide. In high income countries this burden of disease has shifted towards the mostly unvaccinated adult population aged ≥ 65 years, where not only IPD but also NIPD (e.g. respiratory infections) is associated with substantial mortality. We showed that in Belgium some serotypes are more likely to cause IPD, while others are more associated with NIPD and that AMR levels are higher in strains originating from NIPD. These results advocate for the continued surveillance of both IPD and NIPD. In conclusion, the combined serotyping data are imperative in informing policymakers and in turn facilitating the decision of which (adult) pneumococcal vaccination programmes to implement.

## Supplementary Data

1255000 Supplementary Material
