# Supplementary material for: Non-invasive Streptococcus pneumoniae infections are associated with different serotypes than invasive infections, Belgium, 2020 to 2023
Source: Euro Surveill. 2024 Nov 7;29(45):2400108. doi: 10.2807/1560-7917.ES.2024.29.45.2400108 (PMC11544722; doi:10.2807/1560-7917.ES.2024.29.45.2400108)
Supplement: Supplementary Material [file 24-00108_PASSARIS_Supplement.pdf]

This supplementary material is hosted by *Eurosurveillance* as supporting information alongside the article 'Non-invasive Streptococcus pneumoniae infections are associated with different serotypes than invasive infections, Belgium, 2020 to 2023', on behalf of the authors, who remain responsible for the accuracy and appropriateness of the content. The same standards for ethics, copyright, attributions and permissions as for the article apply. Supplements are not edited by *Eurosurveillance* and the journal is not responsible for the maintenance of any links or email addresses provided therein."

SUPPLEMENTARY FIGURES

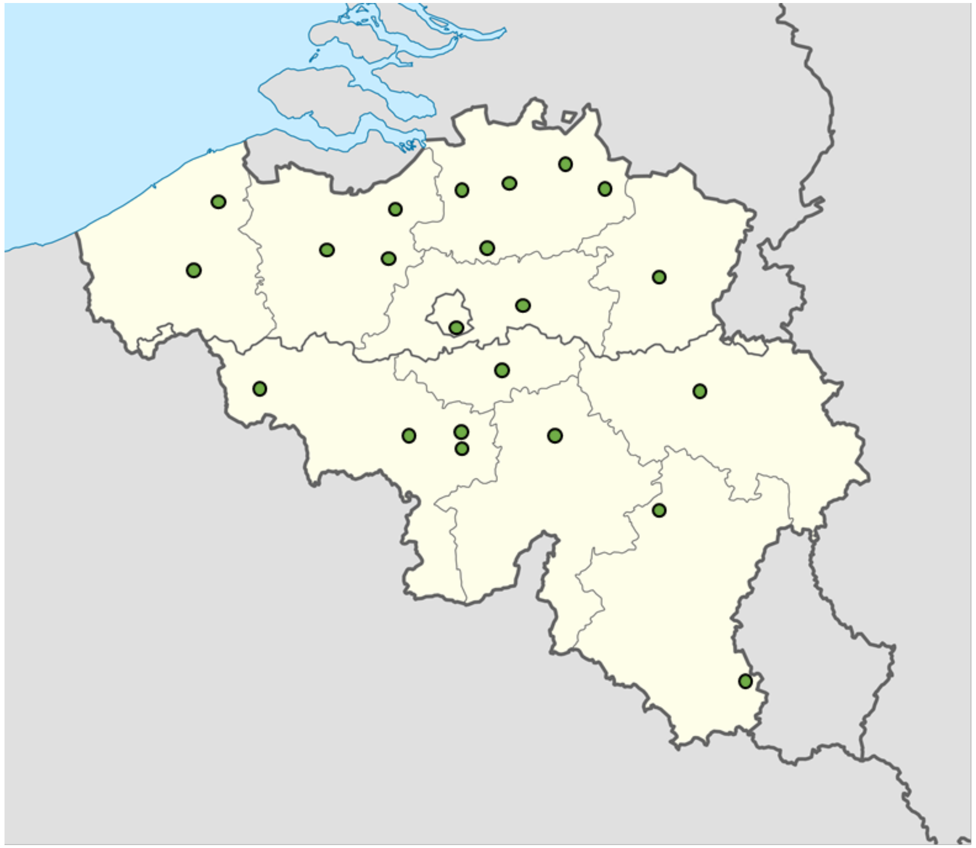

Participating center	City	Region
AZ Nikolaas*	Sint-Niklaas	Flanders
AZ Turnhout*	Turnhout	Flanders
Heilig Hart ziekenhuis Leuven*	Leuven	Flanders
AZ Sint-Jozef*	Malle	Flanders
Heilig Hart ziekenhuis Mol*	Mol	Flanders
ZNA Middelheim	Antwerpen	Flanders
Jessaziekenhuis	Hasselt	Flanders
AZ Delta	Roeselare	Flanders
AZ Sint-Jan	Brugge	Flanders
AZ Jan Palfijn	Gent	Flanders
Imedaziekenhuis	Bonheiden	Flanders
Medisch Labo Medina*	Dendermonde	Flanders
Clinique Notre Dame de Grâce*	Gosselies	Wallonia
Clinique St. Pierre Ottignies*	Ottignies	Wallonia
CHR de Namur*	Namur	Wallonia
CHU de Liège*	Liège	Wallonia
CHR de la Citadelle	Liège	Wallonia
CHU UCL Mont-Godinne	Yvoir	Wallonia
Hôpital de Jolimont	Haine St. Paul	Wallonia
Hôpital de Marche	Marche-en-Famenne	Wallonia
Clinique St. Joseph	Arlon	Wallonia
CHU de Charleroi	Charleroi	Wallonia
Hôpitaux Iris Sud*	Brussels	Brussels

Figure S1: List of all participating centres with their geographical location. Participating centres with an asterisk were only included in the study from March 2021 onwards instead of September 2020.

Classified as ECDC NORMAL

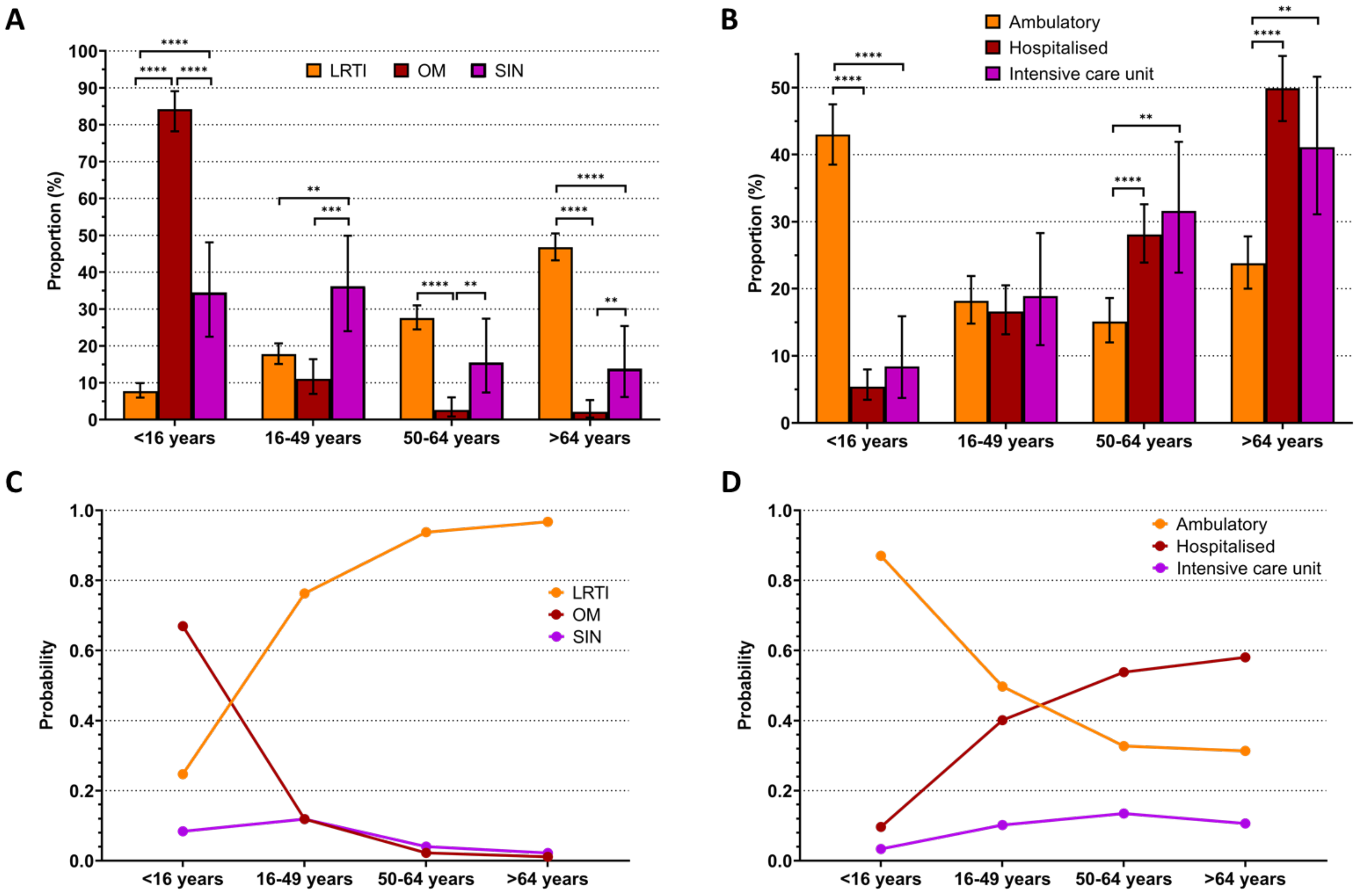

Classified as ECDC NORMAL

Figure S2: Associations between patient age group and (A) clinical diagnosis; (B) type of medical care, and individually calculated probabilities for different age groups being associated with (C) LRTI, OM or sinusitis and (D) ambulatory care, hospitalised care or intensive care. Panel A reveals that patients in the youngest age group (<16 years of age) are significantly more diagnosed with OM when compared to sinusitis and LRTIs in particular, while the exact opposite is observed in all other age groups. Panel B shows that patients in the youngest age group (<16 years of age) received significantly more ambulatory care and were less often hospitalised or admitted to an ICU, while the opposite is found for patients aged 50 years or older. Panel C highlights that the probability of being diagnosed with OM is the highest in the <16 years group (around 70%) and decreases steeply in the adults group (<10%). In contrast, the probability of being diagnosed with a LRTI increases with increasing age and reaches values >90% in patients aged 50 years or older. Panel D indicates that the probability of being hospitalized with NIPD increases with increasing age, reaching almost 60% in patients aged 64 years or older. Statistical significance was assessed with a chi-squared test and exact Fisher's tests were performed for pairwise comparisons (a Bonferroni correction for multiple testing was applied). Error bars represent 95% confidence intervals calculated by the binomial exact method.*=p<0.05, **=p<0.01, ***=p<0.001 and ****=p<0.0001. LRTI, lower respiratory infections; OM, otitis media; SIN, sinusitis. Sample sizes for panels A and C: <16 years (LRTI, n=59; OM, n=160; SIN, n=20), 16-49 years (LRTI, n=135; OM, n=21; SIN, n=21), 50-64 years (LRTI, n=210; OM, n=5; SIN, n=9) and >64 years (LRTI, n=356; OM, n=4; SIN, n=8). Sample sizes for panels B and D: <16 years (Ambulatory, n=208; Hospitalised, n=23; ICU, n=8), 16-49 years (Ambulatory, n=88; Hospitalised, n=71; ICU, n=18), 50-64 years (Ambulatory, n=73; Hospitalised, n=120; ICU, n=30) and >64 years (Ambulatory, n=115; Hospitalised, n=213; ICU, n=39).

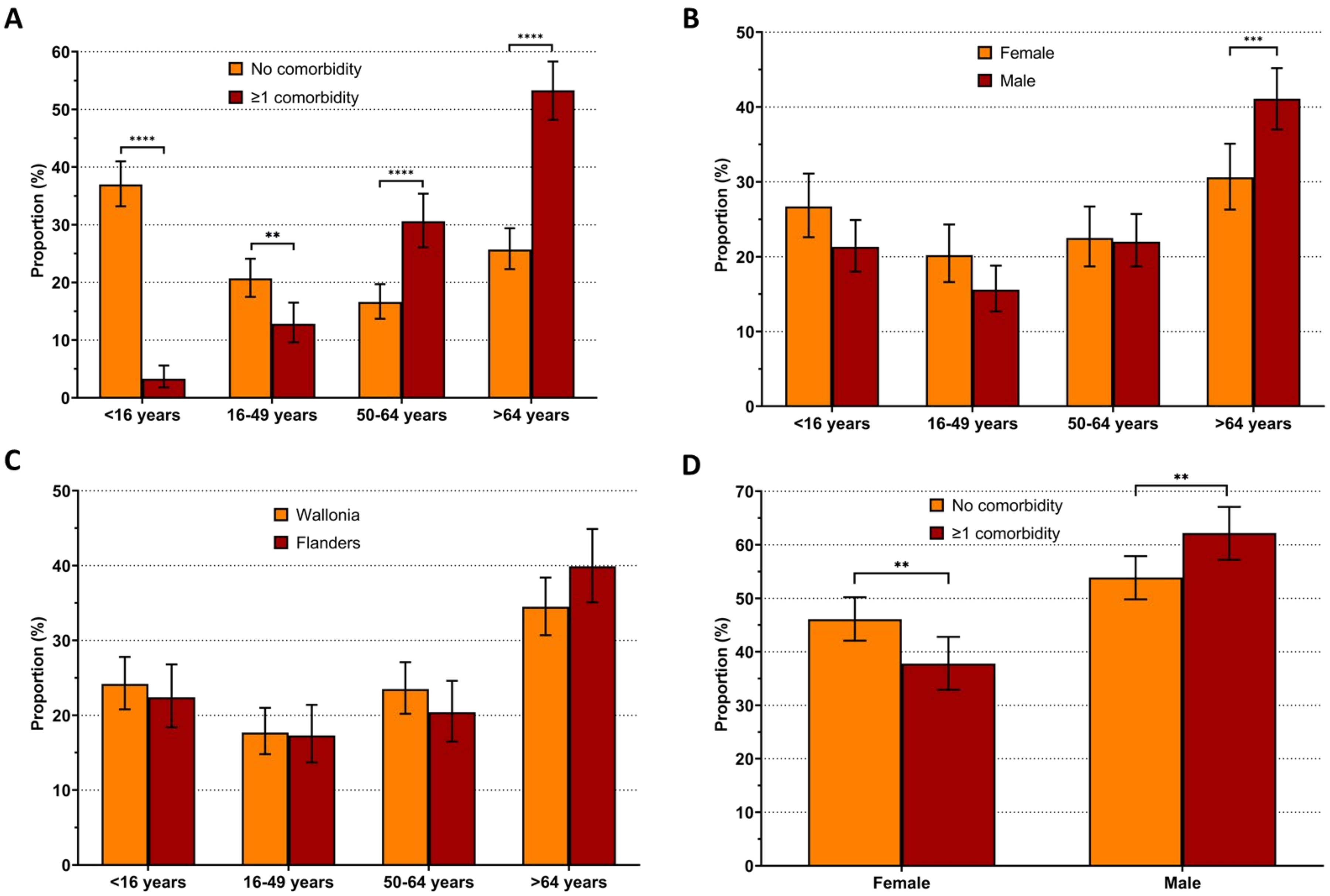
A
Proportion (%)
No comorbidity
≥1 comorbidity
****
**
****
****
<16 years
16-49 years
50-64 years
>64 years
B
Proportion (%)
Female
Male
***
<16 years
16-49 years
50-64 years
>64 years
C
Proportion (%)
Wallonia
Flanders
<16 years
16-49 years
50-64 years
>64 years
D
Proportion (%)
No comorbidity
≥1 comorbidity
**
**
Female
Male

Figure S3: Associations between patient age group and (A) presence of comorbidities; (B) patient sex and (C) geographical region, as well as between (D) patient sex and presence of comorbidities. Panel A indicates that patients aged 49 years or younger are more often found to have no comorbidities at all, while the opposite was true for patients aged 50 years or older. Panel B reveals that patients 64 years or older are significantly more likely to be male. Panel C shows that the age distributions between the two geographical regions (Wallonia and Flanders) are similar. Panel D highlights that male patients are significantly more likely to have at least one comorbidity or immunocompromising condition when compared to female patients. Statistical significance was assessed with Chi-squared tests and exact Fisher's tests were performed for pairwise comparisons. Error bars represent 95% confidence intervals calculated by the binomial exact method. *=p<0.05, **=p<0.01, ***=p<0.001 and ****=p<0.0001. Sample sizes for panel A: <16 years (No comorbidity, n=226; ≥1 comorbidity, n=13), 16-49 years (No comorbidity, n=126; ≥1 comorbidity, n=50), 50-64 years (No comorbidity, n=101; ≥1 comorbidity, n=120) and >64 years (No comorbidity, n=157; ≥1 comorbidity, n=209). Sample sizes for panel B: <16 years (Male, n=122; Female, n=116), 16-49 years (Male, n=89; Female, n=88), 50-64 years (Male, n=126; Female, n=98) and >64 years (Male, n=235; Female, n=133). Sample sizes for panel C: <16 years (Wallonia, n=146; Flanders, n=89), 16-49 years (Wallonia, n=107; Flanders, n=69), 50-64 years (Wallonia, n=142; Flanders, n=81) and >64 years (Wallonia, n=208; Flanders, n=159). Sample sizes for panel D: Female (No comorbidity, n=281; ≥1 comorbidity, n=148) and Male (No comorbidity, n=328; ≥1 comorbidity, n=244).

Classified as ECDC NORMAL

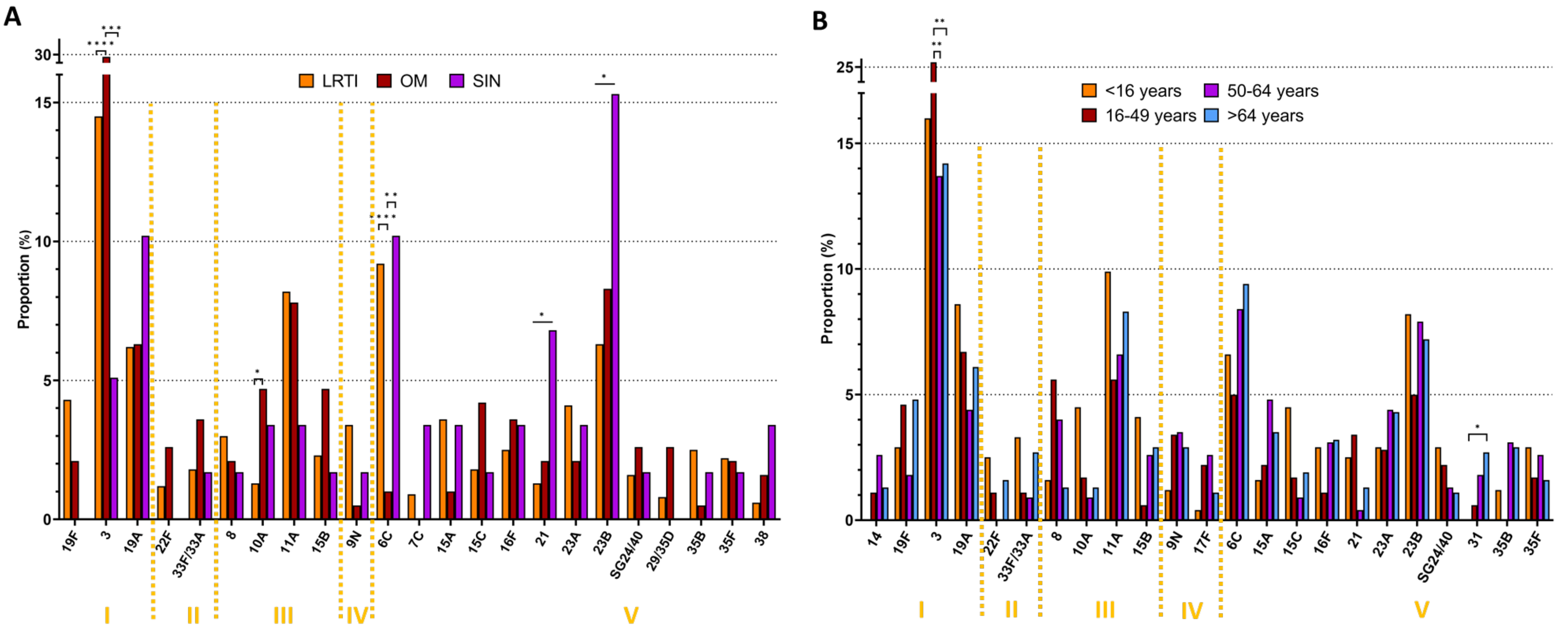

Figure S4: Serotype distribution comparison between (A) the different clinical diagnosis groups (LRTI, n=772; OM, n=192; Sinusitis, n=59) and (B) patient age groups (<16 years, n=243; 16-49 years, n=179; 50-64 years, n=227; >64 years, n=374) shown for specific serotypes with a prevalence of >2% in at least one of the groups. The yellow dotted lines demarcate the serotypes included in different PCVs. I, PCV13 serotypes; II, PCV15-non-PCV13 serotypes; III, PCV20-non-PCV15 serotypes; IV, PPV23 unique serotypes; V, non-PCV20 serotypes. Statistical significance was assessed with exact Fisher's tests (a Bonferroni correction for multiple testing was applied). Error

bars represent 95% confidence intervals calculated by the binomial exact method. *=p<0.05, **=p<0.01, ***=p<0.001 and ****=p<0.0001.

LRTI, lower respiratory infection; OM, otitis media; SIN, sinusitis.

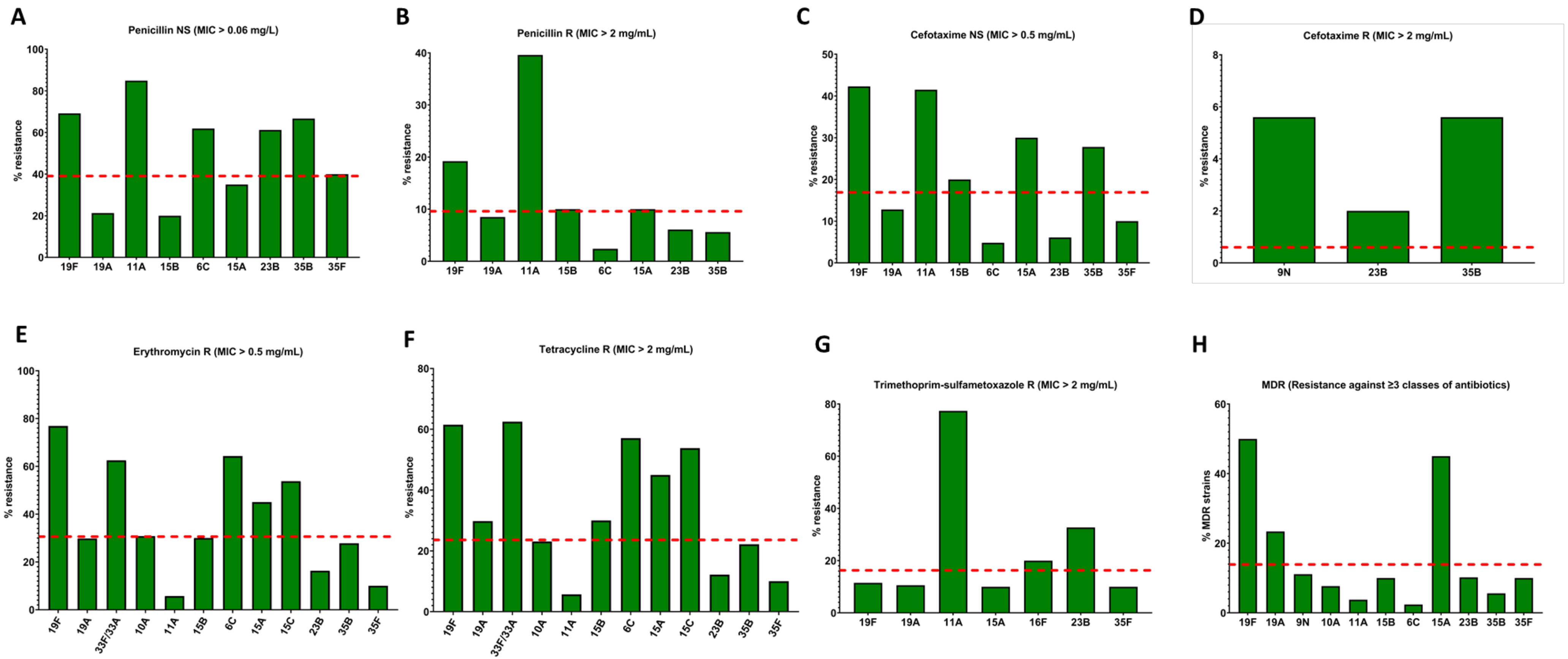

Figure S5: AMR resistance in the NIPD population (2020-2023, n=539) in relation with serotype. Panels A to G show resistance against different antimicrobials and antimicrobial concentrations for relevant serotypes. Only serotypes with a prevalence of ≥2% in the NIPD population were included in the analysis. The red dotted line shows the average resistance of the entire population against the specific antimicrobial.

Classified as ECDC NORMAL

SUPPLEMENTARY TABLES

Table S1: Serotype distribution of the NIPD population in Belgium (2020-2023).

Serotype	number of strains	%	Vaccine
3	170	16.6	PCV13
11A	80	7.8	PCV20
6C	79	7.7	NVT
23B	74	7.2	NVT
19A	66	6.5	PCV13
23A	38	3.7	NVT
19F	37	3.6	PCV13
15A	32	3.1	NVT
9N	28	2.7	PPV23
16F	28	2.7	NVT
8	28	2.7	PCV20
15B	28	2.7	PCV20
15C	23	2.2	NVT
35F	22	2.2	NVT
33F/33A	22	2.2	PCV15
35B	21	2.1	NVT
10A	21	2.1	PCV20
21	18	1.8	NVT
SG24/40	18	1.8	NVT
17F	15	1.5	PPV23
31	15	1.5	NVT
22F	14	1.4	PCV15
14	13	1.3	PCV13
38	10	1.0	NVT
7C	9	0.9	NVT
29/35D	9	0.9	NVT
4	08	0.8	PCV13
34	6	0.6	NVT
20	5	0.5	PPV23
13	3	0.3	NVT
6B	2	0.2	PCV13
18C	2	0.2	PCV13
6A	2	0.2	PCV13
6D	2	0.2	NVT
12F	2	0.2	PCV20
7F	1	0.1	PCV13
23F	1	0.1	PCV13
9V	1	0.1	PCV13
unknown	70	6.8	

Classified as ECDC NORMAL

Table S2: AMR of NIPD isolates for the different tested antimicrobials in this study (n=539).

	Number of strains	%
**Penicillins**		
penicillin MIC > 0.06 mg/L	211	39.1
penicillin MIC > 2 mg/L	52	9.6
amoxicillin MIC > 1 mg/L	87	16.1
amoxicillin-clavulanic acid MIC > 2 mg/L	58	10.8
**Cephalosporins**		
Cefotaxime MIC > 0.5 mg/L	91	16.9
Cefotaxime MIC > 2 mg/L	3	0.6
Cefuroxime MIC > 0.5 mg/L	134	24.9
**Carbapenems**		
Imipenem MIC > 2 mg/L	0	0.0
Meropenem MIC > 2 mg/L	0	0.0
**Fluoroquinolones**		
Levofloxacin MIC > 2 mg/L	2	0.4
Moxifloxacin MIC > 0.5 mg/L	2	0.4
**Macrolides, lincosamides and streptogramins**		
Erythromycin MIC > 0.5 mg/L	165	30.6
Clindamycin MIC > 0.5 mg/L	132	24.5
**Tetracyclines**		
Tetracycline MIC > 2 mg/L	127	23.6
**Miscellaneous**		
Trimethoprim-sulfamethoxazole MIC > 2 mg/L	88	16.3
**MDR (Resistance against ≥3 classes of antibiotics)**	75	13.9
**XDR (Resistance against ≥5 classes of antibiotics)**	0	0.0

Classified as ECDC NORMAL

Table S3: Comparison of MIC^{50} and MIC^{90} values between the NIPD population (2007-2008, n=448) and the NIPD population (2020-2023, n=539) for relevant β-lactam antimicrobials. PEN, penicillin; FOT, cefotaxime; AMOX, amoxicillin and IMI, imipenem.

	NIPD 2007-2008	NIPD 2020-2023
**MIC^{50} PEN**	0.015 mg/L	0.03 mg/L
**MIC^{50} FOT**	0.015 mg/L	0.03 mg/L

**MIC^{90} PEN**	0.25 mg/L	2 mg/L
**MIC^{90} FOT**	0.25 mg/L	1 mg/L
**MIC^{90} AMOX**	0.06 mg/L	4 mg/L
**MIC^{90} IMI**	0.03 mg/L	0.5 mg/L

Classified as ECDC NORMAL
